# Supplementary material for: Residual microglia following short‐term PLX5622 treatment in 5xFAD mice exhibit diminished NLRP3 inflammasome and mTOR signaling, and enhanced autophagy
Source: Aging Cell. 2024 Nov 21;24(2):e14398. doi: 10.1111/acel.14398 (PMC11822669; doi:10.1111/acel.14398)
Supplement: Supplementary file 1 — Appendix S1. [file ACEL-24-e14398-s001.pdf]

Supplemental File

Residual Microglia Following Short-term PLX5622 Treatment in 5xFAD Mice Exhibit Diminished NLRP3 Inflammasome and mTOR Signaling, and Enhanced Autophagy

Maheedhar Kodali*, Leelavathi N. Madhu*, Yogish Somayaji, Sahithi Attaluri, Charles Huard, Prashanta Kumar Panda, Goutham Shankar, Shama Rao, Bing Shuai, Jenny J. Gonzalez, Chris Oake, Catherine Hering, Roshni Sara Babu, Sanya Kotian and Ashok K. Shetty[§]

**** Co-first authors**

Institute for Regenerative Medicine, Department of Cell Biology and Genetics, Texas A&M University Health Science Center School of Medicine, College Station, Texas, USA

Supplemental Figure 1

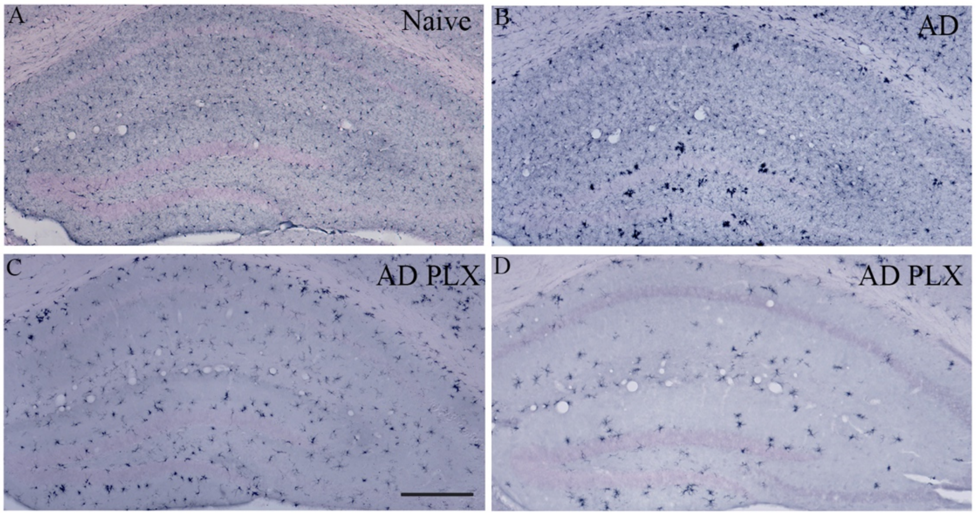

Supplemental Figure 1: Ten days of CSF1R inhibition in 3-month-old 5xFAD mice caused a partial depletion of microglia in the hippocampus. Figures A–D illustrate examples of lower magnification images of hippocampal IBA1+ microglia for naïve (A), AD (B), and AD+PLX (C, D) groups.

Supplemental Figure 2

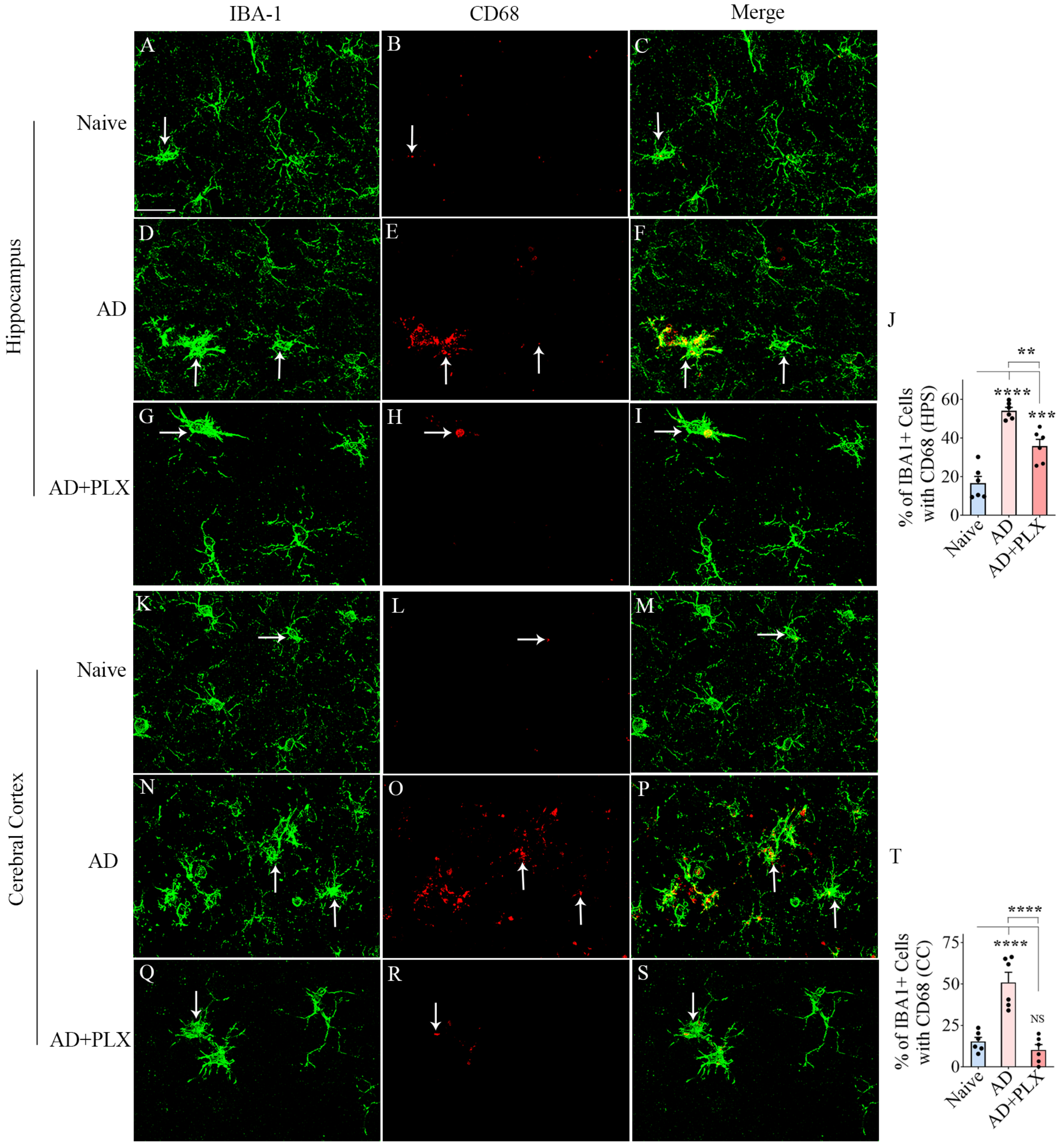

Supplemental Figure 2: Ten days of CSF1R inhibition in 3-month-old 5xFAD mice reduced the percentages of microglia expressing CD68 in the hippocampus and cerebral cortex. Figures A–I illustrate IBA-1+ microglia (green) displaying CD68+ structures (red) from the hippocampal CA3 subfield of naive (A-C), AD (D-F), and AD+PLX (G-I) groups. Figures K-S illustrate IBA-1+ microglia (green) displaying CD68+ structures (red) from the cerebral cortex of naive (K-M), AD (N-P), and AD+PLX (Q-S) groups. The bar charts in J and T compare the percentages of IBA-1+ microglia with CD68 in the hippocampus (J) and cerebral cortex (T). Scale bar, A-R = 10 μm; *, $p < 0.05$; **, $p < 0.01$; ***, $p < 0.001$; ****, $p < 0.0001$; NS, not significant.

Supplemental Figure 3

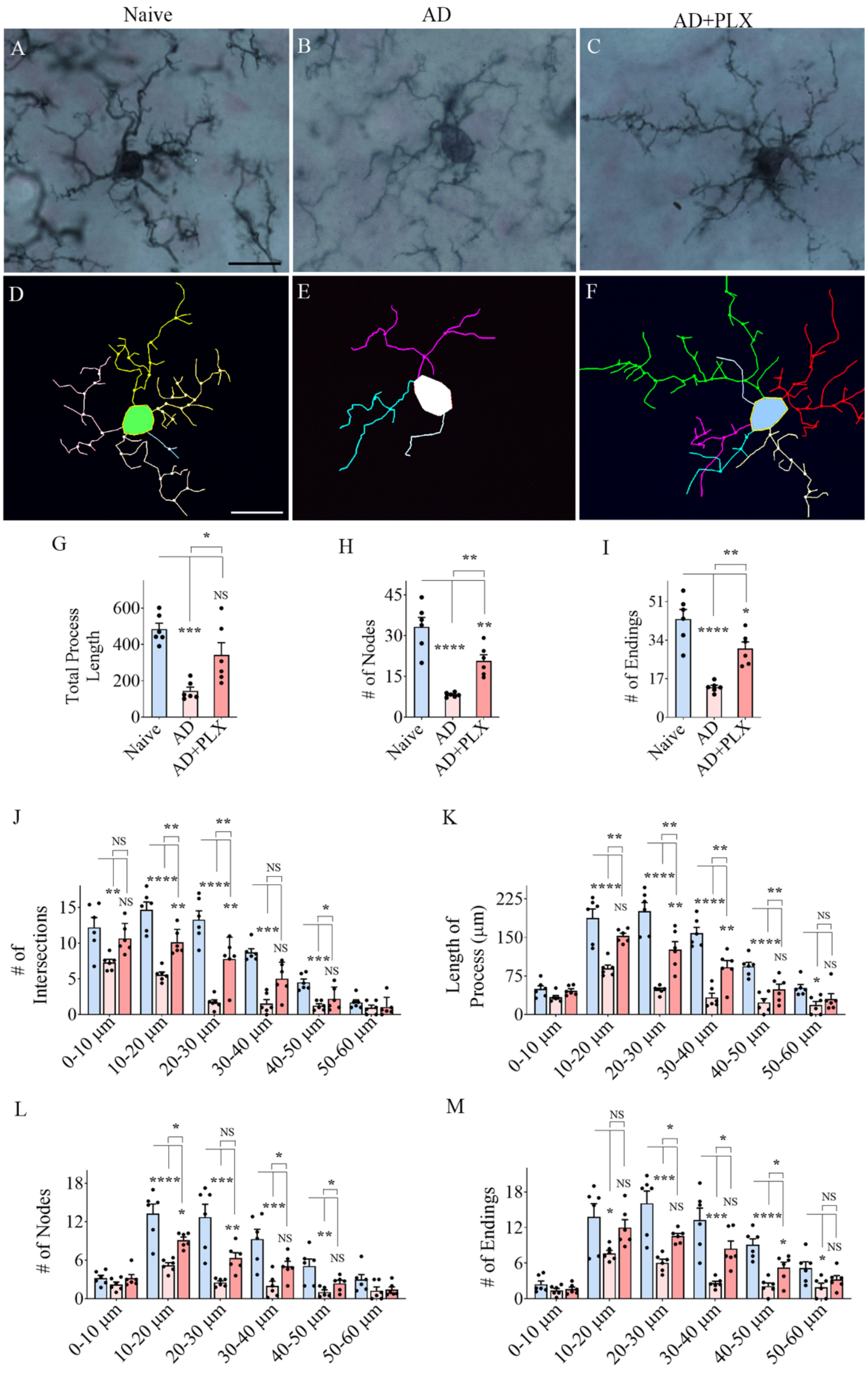

Supplemental Figure 3: Residual microglia following CSF1R inhibition displayed highly branching and ramified processes in the cerebral cortex of 5xFAD mice. A-F shows representative examples of microglial morphology traced with Neurolucida from the cerebral cortex of naïve (A, D), AD (B, E), and AD+PLX (C, F) groups. The bar charts G-I compare the various morphometric measures of microglia between naïve, AD and AD+PLX groups, which include the total process length (G), the number of nodes (H), and the number of process endings (I). The bar charts J-M compare the number of intersections (J), total process length (K), the number of nodes (L), and the number of process endings (M) between naïve, AD and AD+PLX groups at 0–10 μm, 10–20 μm, 20–30 μm, 30–40 μm, 40–50 μm, and 50–60 μm distances from the soma. Scale bar, A-F = 12.5 μm; *, $p < 0.05$; **, $p < 0.01$; ***, $p < 0.001$; ****, $p<0.0001$ NS, not significant.

Supplemental Figure 4

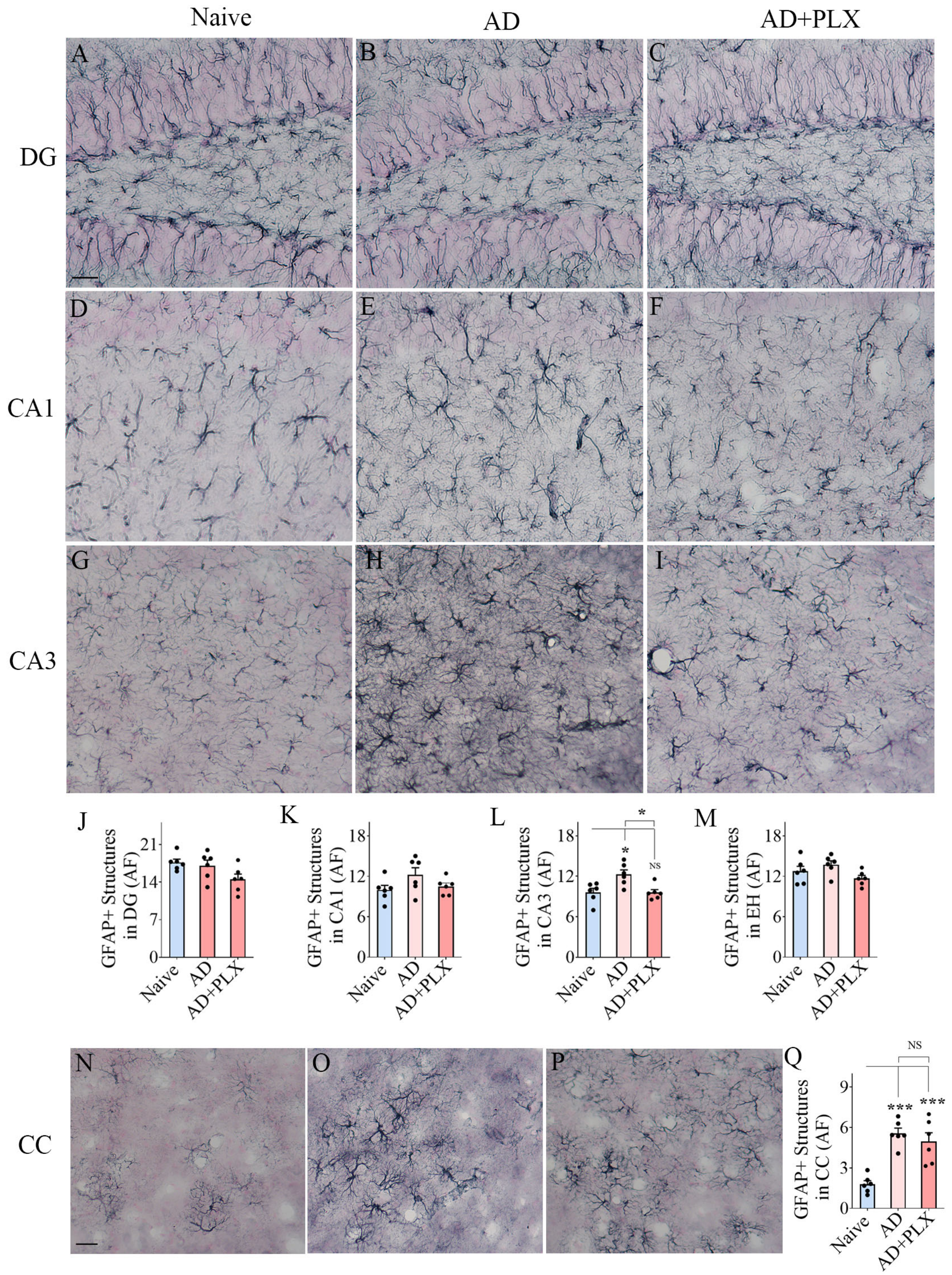

Supplemental Figure 4: Transient CSF1R inhibition reduced astrocyte hypertrophy only in the CA3 region of the hippocampus in 5xFAD mice. Figures A-I illustrate representative examples of GFAP+ astrocytes in the DG (A-C), CA1 (D-F), and CA3 (G-I) regions of the hippocampus of naïve (A, D, G), AD (B, E, H), and

AD+PLX (C, F, I) groups. Bar charts J-M compare the AF of GFAP+ structures in DG (J), CA1 (K), CA3 (L), and the entire hippocampus (EH, M) across the groups. Figures N-P illustrate representative examples of GFAP+ astrocytes in the cerebral cortex (CC, N-P) between naïve (N), AD (O) and AD-PLX groups (P). The bar chart Q compares the AF of GFAP+ structures in the cerebral cortex (CC) across groups. Scale bar, A-I and N-P = 40 µm. **, $p < 0.05$; ***, $p < 0.001$; NS, not significant.*

Supplemental Figure 5

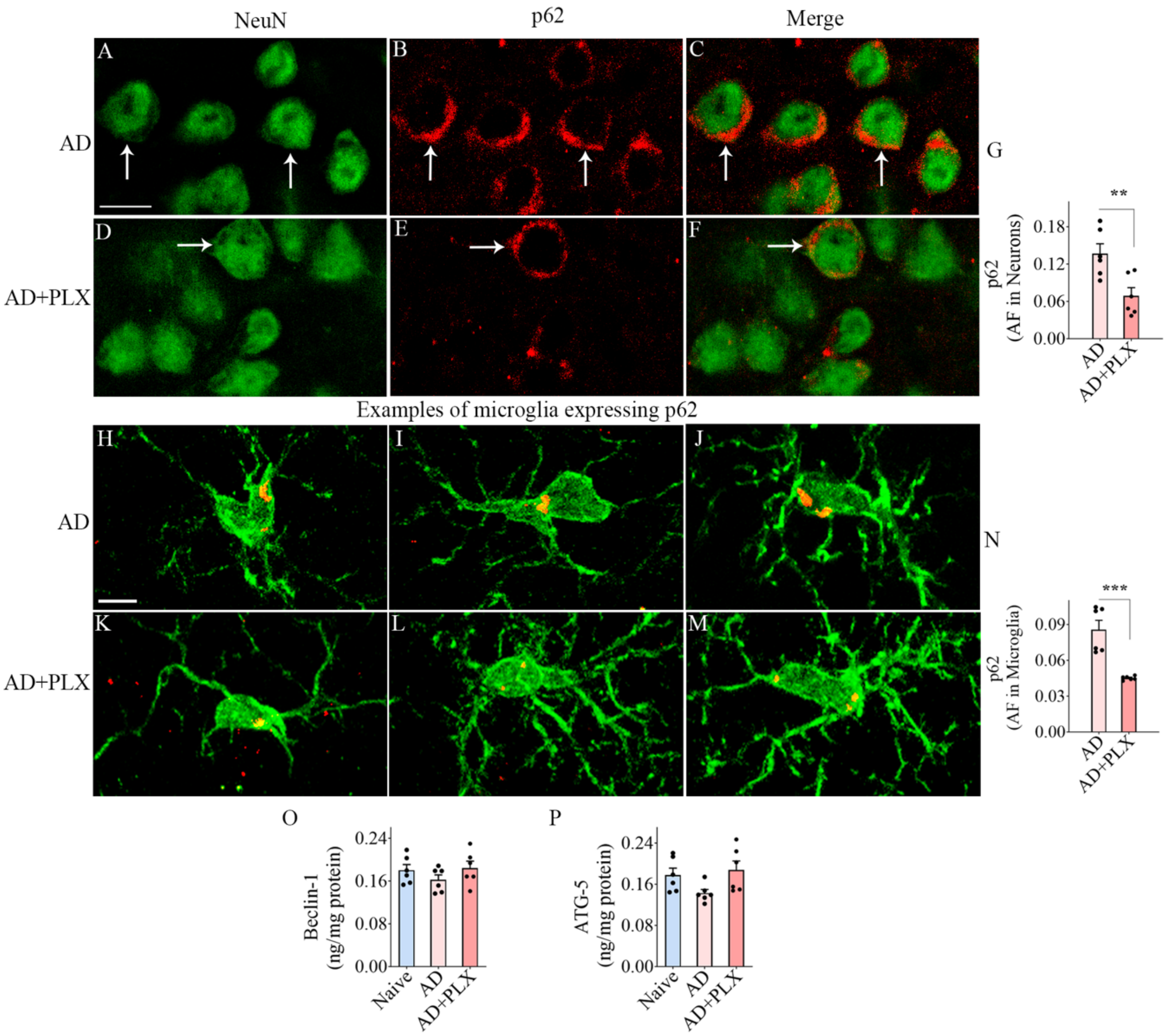

Supplemental Figure 5: Ten days of CSF1R inhibition in 5xFAD mice modestly enhanced autophagy in the cortical neurons and microglia. Figures A-F show examples of neurons expressing p62 (a marker of autophagic flux) in the CA3 subregion of the hippocampus of AD (A-C) and AD+PLX (D-F) groups. The bar chart G compares the area fraction (AF) of p62 in NeuN+ neurons between AD and AD+PLX groups. Figures H-M illustrate microglia expressing p62 from AD (H-J) and AD+PLX groups (K-M) groups. The bar chart N compares the AF of p62 in IBA-1+ microglia between AD and AD+PLX groups in the hippocampus. The bar charts O-P compare the concentration of autophagy-related proteins, beclin-1 (O), and autophagy-related 5 (ATG-5, P), between naïve, AD and AD+PLX groups. Scale bar, A-F = 10 µm, H-M = 2.5 µm *, p<0.05; **, p<0.01; ****, p < 0.0001;   NS, not significant.

Supplemental Figure 6

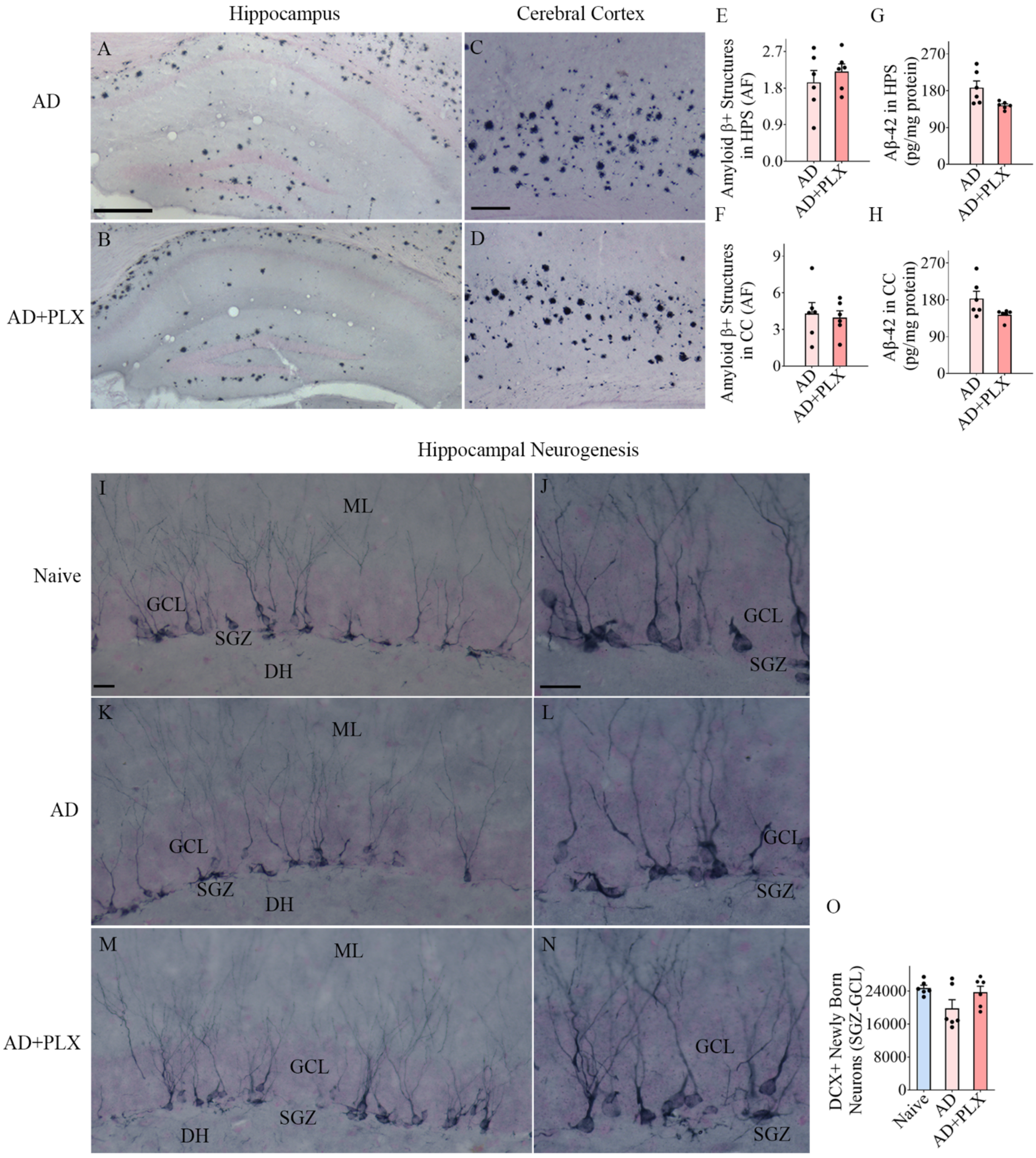

Supplemental Figure 6: Ten days of CSF1R inhibition in 5xFAD mice did not alter the amyloid-beta plaques in the hippocampus and cerebral cortex and hippocampal neurogenesis. Figures A–D illustrate examples of amyloid beta (Aβ) plaques in the hippocampus (A, B) and the cerebral cortex (C, D) from AD (A, C) and AD+PLX (B, D) groups. The bar charts E-F compare the area fraction of Aβ plaques between AD

and AD+PLX groups from the hippocampus (E) and the cerebral cortex (F). Scale bar, A-B = 250 μm, C-D=125 μm. Bar charts G-H compare Aβ-42 concentrations between AD and AD+PLX groups in the hippocampus (G) and the cerebral cortex (H). Figures I-N illustrate examples of doublecortin-positive (DCX+) newly born neurons from naïve (I-J), AD (K-L), and AD+PLX (M-N) groups. Bar chart O compares the number of DCX+ neurons between naïve, AD, and AD+PLX groups. GCL, granule cell layer; SGZ, subgranular zone; DH, dentate hilus; ML, molecular layer. Scale bar, I-N = 20 μm.

Supplemental Table 1: The list of primary and secondary antibodies employed in the study

Primary Antibodies			
**Protein**	**Antibody**	**Company name, Country**	**Dilution**
Iba1	goat anti-IBA-1	Abcam, USA	1:1000
Iba1	rabbit anti-IBA-1	Abcam, USA	1:1000
Amyloid beta	rabbit anti-Aβ42	Invitrogen, USA	1:500
Clec7a	rat anti-dectin-1 (Clec7a)	InvivoGen, USA	1:100
NLRP3	goat anti-NLRP3	Millipore, USA	1:500
ASC	mouse anti-ASC	Santa Cruz, USA	1:500
CD68	mouse anti-CD 68	Biorad, USA	1:500
GFAP	rabbit anti-GFAP	DAKO, USA	1:2000
NeuN	mouse anti-NeuN	Millipore, USA	1:1000
pS6	rabbit anti-phospho-S6	Cell Signalling, USA	1:200
p62	guinea pig anti-p62	Progen, Germany	1:500
Doublecortin	rabbit anti-DCX	Synaptic Systems, Germany	1:1000

Secondary Antibodies		
**Antibody**	**Company name**	**Dilution**
anti-rabbit IgG	Vector Labs, USA	1:200
anti-goat IgG	Vector Labs, USA	1:200
donkey anti-mouse IgG tagged with Alexa Fluor 488	Invitrogen, USA	1:200
donkey anti-mouse IgG tagged with Alexa Fluor 594	Invitrogen¸ USA	1:200
donkey anti-mouse IgG tagged with Alexa Fluor 405	Invitrogen¸ USA	1:200
donkey anti-goat IgG tagged with Alexa Fluor 488	Invitrogen¸ USA	1:200
donkey anti-goat IgG tagged with Alexa Fluor 594	Invitrogen¸ USA	1:200
donkey anti-rabbit IgG tagged with Alexa Fluor 405	Invitrogen¸ USA	1:200
donkey anti-rabbit IgG tagged with Alexa Fluor 488	Invitrogen¸ USA	1:200
donkey anti-rabbit IgG tagged with Alexa Fluor 594	Invitrogen¸ USA	1:200
donkey anti-guinea pig Alexa Fluor 594	Invitrogen¸ USA	1:200
donkey anti-rat IgG tagged with Alexa Fluor 594	Invitrogen¸ USA	1:200
